# Signaling Pathways and Therapeutic Strategies in Advanced Basal Cell Carcinoma

**DOI:** 10.3390/cells12212534

**Published:** 2023-10-27

**Authors:** Giulia Vallini, Laura Calabrese, Costanza Canino, Emanuele Trovato, Stefano Gentileschi, Pietro Rubegni, Linda Tognetti

**Affiliations:** 1 Department of Medical Biotechnologies, University of Siena, 53100 Siena, Italy; 2 Department of Medical, Surgical and Neurological Sciences, Division of Dermatology, University of Siena, 53100 Siena, Italy; laura.calabrese@unisi.it (L.C.); emanuele.trovato@unisi.it (E.T.); pietro.rubegni@unisi.it (P.R.); linda.tognetti@dbm.unisi.it (L.T.); 3 Institute of Dermatology, Catholic University of the Sacred Heart, 00168 Rome, Italy; 4 Department of Haematology, Oncology and Cell and Gene Therapy, IRCCS Bambino Gesù Children’s Hospital, 00165 Rome, Italy; costanza.canino@opbg.net; 5 Department of Medical, Surgical and Neurological Sciences, Division of Rheumatology, University of Siena, 53100 Siena, Italy; stefano.gentileschi@unisi.it

**Keywords:** basal cell carcinoma, Hedgehog-GLI pathway, BET, immunotherapy

## Abstract

Non-melanoma skin cancers (NMSCs) are the most common human neoplasms world-wide. In detail, basal cell carcinoma (BCC) is the most frequent malignancy in the fair-skinned population. The incidence of BCC remains difficult to assess due to the poor registration practice; however, it has been increasing in the last few years. Approximately, 85% of sporadic BCCs carry mutations in Hedgehog pathway genes, especially in PTCH, SUFU and SMO genes, which lead to the aberrant activation of GLI transcriptional factors, typically silent in cells of adult individuals. The management of advanced BCC (aBCC), both metastatic (mBCC) and locally advanced BCC (laBCC), not candidates for surgical excision or radiotherapy, remains challenging. The discovery of mutations in the Hh signaling pathway has paved the way for the development of Hh pathway inhibiting agents, such as vismodegib and sonidegib, which have represented a breakthrough in the aBCC management. However, the use of these agents is limited by the frequent occurrence of adverse events or the development of drug resistance. In this review, we thoroughly describe the current knowledge regarding the available options for the pharmacological management of aBCCs and provide a forward-looking update on novel therapeutic strategies that could enrich the therapeutic armamentarium of BCC in the near future.

## 1. Introduction

Non-melanoma skin cancers (NMSCs) are the most common human cancer worldwide. Basal cell carcinoma (BCC) and cutaneous squamous cell carcinoma (cSCC) represent 99% of NMSC [1].

In detail, BCC is the most frequent malignancy in the fair-skinned population. Metastatic activity is a rare event in BCC history, as the association with death; however, the progressive growth of the tumor can be invasive and destructive [2].

At first, BCC was thought to originate from bulge stem cells in the hair follicle; lately, a study carried out in mice refuted this hypothesis and proposed that neoplastic cells actually arise from long-term resident progenitor cells of the interfollicular epidermis and the upper infundibulum [3].

The aim of this review is to thoroughly describe the currently available options for the pharmacological management of aBCCs and to highlight promising therapeutic strategies that could enrich the therapeutic armamentarium of BCC in the future. Moreover, the present review provides an overview of the epidemiology, clinical features and pathogenesis of BCC, with a focus on the Hedgehog signaling cascade, a major pathway involved in BCC onset. Indeed, deepening the knowledge of the molecular mechanisms underlying the pathogenesis of BCC is essential to develop new therapeutic strategies that can overcome the limits of the current pharmacological treatments.

## 2. Epidemiology

The incidence of BCC varies widely depending on the country’s geographic latitude and on the predominant phototype of the population [4,5], though it remains difficult to assess due to the poor registration practice and the non-distinction in registries between different NMSCs. The highest incidence rate in the world has been registered in Australia, even though the most recent study, reporting an age-standardized rate of 884/100,000 persons, dates back to 2002 [6]. This was 10 times the rate of the UK in the same period [7]. In the USA the latest study stated that in 2012, 5.4 million cases were diagnosed among 3.3 million people with an age-adjusted procedure rate of 3280/100,000 persons [8]. There are no clear data about the comprehensive incidence of BCC in South America. A report from São Paolo Hospital in Brazil estimated an incidence rate of 36 cases per 100,000 patients/year [9]. According to Basset-Seguin (2020), the incidence rate of BCC in Europe ranges from 76.21/100,000 person-years in the UK to 157 per 100,000 person-years in 2009 in the Netherlands [10], basically conforming to a systematic review from 14 European countries reporting an incidence rate between 24 and 170/100,000 during the first decade of 21st century [4]. Regarding the incidence of BCC in Africa, a thirty-year retrospective study (1968–1997) in Kenya showed a mean annual incidence rate per 100,000 persons of 0.065. As far as we know, very little information is available about the epidemiology of BCC in the Asian population. A study conducted from 1968 to 1997 on Chinese, Malay and Indian people reported age-standardized incidence rates of 2.6/100,000 person in the first five years and 5.5/100,000 in the last five years with an increase in incidence of 3% annually [11].

Globally, the incidence rate of BCC in the last decades continues to increase.

## 3. Pathogenesis

### 3.1. Environmental Factors

The significant increase in BCC’s incidence may be partially explained by people’s lifestyle changes such as inappropriate ultraviolet (UV) exposure in short-term recreational tanning salons [12,13] as well as environmental changes like ozone depletion, responsible for filtering most of the UV-B. Particularly, as stratospheric ozone decreases, shorter wavelengths (280–320 nm), the most effective for photo-carcinogenesis, reach ground level [14]. Indeed, it is well established that exposure to UV radiation is the main cause of skin cancer and can induce deleterious mutations leading to the activation of proto-oncogenes, such as the RAS family, or to the loss of function of oncosuppressor genes, such as p53 and PTCH, implicated in the pathogenesis of BCC [15]. Typical UV-signature mutations, C → T transition at dipyrimidine sites and CC → TT tandem mutation, of p53 and PTCH genes are commonly detected in BCC [16,17,18].

However, if the literature data suggest a strong correlation between the risk of developing BCCs and fair pigmentary characteristics as well as frequent sunburn episodes experienced in childhood [19,20], the real impact of cumulative UV exposure is still unclear [21].

Another much-investigated risk factor is immunosuppression, despite its role in BCC pathogenesis is still controversial. Transplanted patients display a greater risk of developing skin malignancies (approximately 100-fold by 5 years after transplantation), probably due to prolonged immunosuppressive therapy [22]. Although the most common NMSC in this setting is cSCC, the risk of developing a BCC in organ transplant patients is still increased from 6-fold to 16-fold compared to the general population [23,24].

Moreover, epidemiological studies describe exposure to ionizing radiations (e.g., from X-rays, radiotherapy, and atomic bombs) as an important environmental risk factor for BCC development [25].

### 3.2. Genetic Features

BCC is strongly associated with epidermal keratinocyte genetic alterations, showing one of the highest tumor mutational burdens (TMB) among all cancer types (47.3 mutations/Mb) [26].

A small percentage (0.4%) of BCCs (2% among patients aged under 45) is associated with nevoid basal cell carcinoma syndrome (NBCCS), also known as Gorlin syndrome [27,28]. This rare genetic disorder is characterized by a predisposition to develop multiple BCCs, epidermal cysts of the skin, odontogenic keratocysts of the jaws, palmoplantar pits, calcified dural folds, and other neoplasms (e.g., medulloblastoma and ovarian fibroma), as well as various skeletal anomalies. Studies conducted on Gorlin syndrome led to the identification of a pathogenic germline mutation of protein receptor Patched (PTCH) 1, a key protein of the Hedgehog (Hh) pathway [29]. The function of the Hh signaling pathway is to regulate proliferation and differentiation during embryonic development, and it is generally turned off in adulthood, except for its role in tissue homeostasis and stem cell maintenance. An aberrant activation of Hh signaling plays a well-established role in human neoplasms [30]. While the significance of PTCH1 mutations in NBCCS pathogenesis is well established, the role of the homolog PTCH2 is uncertain. While PTCH1 is principally expressed in mesenchymal cells, PTCH2 is expressed in skin and testicular epithelial cells [31]. PTCH2 is a functional Hh receptor, able to cooperate with PTCH1 in the negative regulation of Smoothened (SMO), a seven-pass transmembrane protein with similarities to G protein-coupled receptors, involved itself in the Hh signaling cascade [32]. In 2008, a mutation (2157G→A) in exon 15 of PCTH2 has been reported in a Chinese family with some NBCCS phenotypes [33]. In 2013, a case of a patient with a NBBCS diagnosis and a frameshift mutation (1172_1173delCT) in this gene was described [34] and, lately, a case of a family with a novel PTCH2 mutation in exon 21 (3347C→T) was reported [35]. In all these reports, the patients did not exhibit a typical Gorlin syndrome phenotype and Smith and Evans highlighted the uncertain pathogenetic significance of the described mutations, prompting that PTCH2 might not be a bona fide Gorlin syndrome predisposition gene [36].

In a similar way, sporadic BCCs often carry aberrant activations of the Hh pathway resulting from genetic inactivation of PTCH1 or PTCH2, as well as from activating mutations in SMO or inactivating mutations in Suppressor of Fused (SUFU), which are both key Hh signal transducers [37,38,39]. Approximately, 85% of sporadic BCCs carry mutations in Hh pathway genes; 73% of them involve loss of function (LOF) mutations in PTCH, 20% gain-of-functions (GOF) mutations in SMO, 8% LOF mutations in SUFU. In addition, BCC can harbor mutations in other important cancer-related genes, such as TP53, members of the RAS family, MYCN, PPP6C, PTPN14, STK19, and LATS1 [40]. Some of these, like the RAS-RAF-MEK-ERK pathway, have a positive role in the activation of the Hh pathway in a non-canonical manner [41].

In detail, ‘Hedgehog’ proteins are hydrophobic ligands with three homologs in humans: Sonic hedgehog (SHh), Indian hedgehog (IHh) and Desert hedgehog (DHh). Hh signaling is dependent on the primary cilium, a microtubule-based, membrane-enclosed structure and is mediated by the action of GLI 1/2/3 transcriptional factors [42]. GLI 2/3 can act as both activators or repressors, depending on whether they are in full length or in the cleavage form, respectively. They are the primary mediators of the Hh pathway and in stimulated cells can activate the expression of GLI1, a target gene of Hh signaling, thus establishing a positive feedback loop [43]. 

Upon binding of Hh ligands, PTCH releases the inhibition on SMO. SMO is phosphorylated by casein kinase 1 α (CK1α) and G protein-coupled receptor kinase 2 (GRK2) and this promotes the accumulation of SMO in the primary cilium and its activation. Activated SMO, in turn, binds Suppressor of Fused (SUFU), a crucial negative regulator of Hh signaling activity, thus preventing the proteolytic processing of the Glioma-associated oncogene homolog transcription factors 2 (GLI2), that translocates in the nucleus activating the transcription of Hh target genes, including GLI1 [44,45].

**Figure 1:**
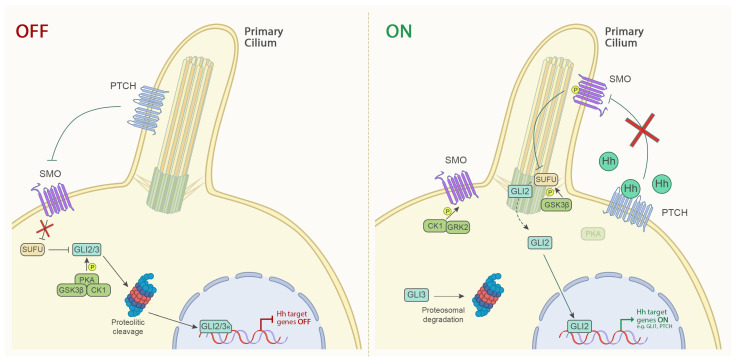
The Canonical Hedgehog Signaling Pathway. In the absence of a Hh ligand, PTCH inhibits SMO and SUFU binds to GLI transcriptional factors. PKA, CK1, and GSK3β, in turn, phosphorylate GLI transcriptional factors GLI2 and GLI3, leading them to proteolytic cleavage. Upon binding of a ligand to PTCH, the inhibition of SMO is relieved. SMO is phosphorylated by CK1α and GRK2, promoting its accumulation in the cilium. Consequently, SUFU, phosphorylated by GSK3β, releases GLI2 that translocates in the nucleus and activates the Hh target genes. To note, the dual role of GSK3β, which can act as both a promoter and a suppressor of the Hh pathway.

In the absence of the ligand, PATCH inhibits SMO. When SMO is inactive, SUFU binds GLI transcriptional factors. Subsequently, CK1α, protein kinase A (PKA) and glycogen synthase kinase 3 β (GSK3β) phosphorylate GLI 2/3 that are truncated into the repressor form, thus inhibiting transcription of Hh target genes (Figure 1) [46].

GSK3β, identified in 2007 by Takenada et al. as a GSK3 isoform [47], seems to have a dual role in the Hh-Gli signaling pathway. On one hand, it mediates the phosphorylation of GLI proteins, tagging them for degradation and processing into repressor forms in the inactive state; on the other hand, it binds SUFU in ligand-stimulated cells and enables the release of GLI from the complex [48].

Notably, GLI family oncogenes have been implicated in the oncogenesis of several tumors. GLI proteins can stimulate cell proliferation by the transcriptional activation of key regulators of G1/S phase progression, can induce the up-regulation of the bcl-2 anti-apoptotic proto-oncogene, and enhance the metastatic spread by activation of EMT-promoting factors such as Snail [49].

## 4. Clinical Features

**Figure 2:**
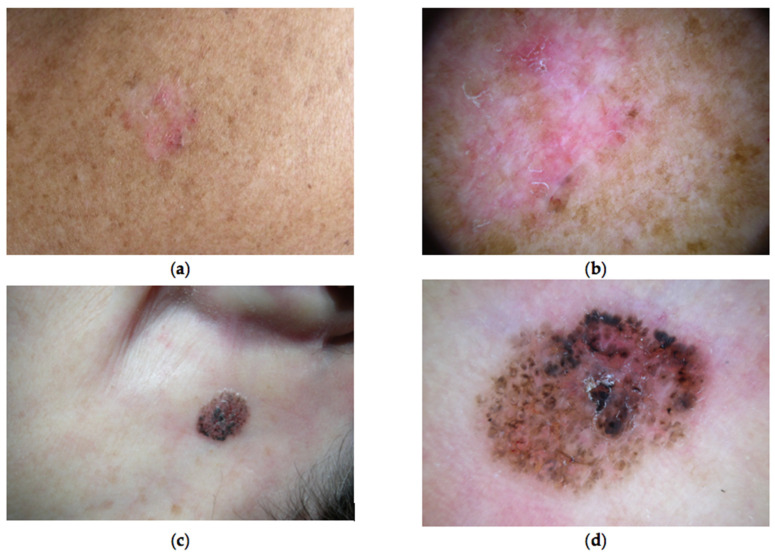
Clinical and dermoscopic (polarized, 20× enlargement) appearance of two BCC types. A superficial BCC of the back in a 73 years-old man, consisting in a flat erythematous macule with undefined margins (**a**): dermoscopic examination highlights the presence of in-focus vessels with arborizing pattern in some areas, brown dots and focal peripheral scaling (**b**). A nodular-type pigmented BCC of the neck in a 62 years-old woman (**c**): dermoscopy reveals dark brown large globules corresponding to ovoid nests, small dark brown structures corresponding to spoke-wheel areas and light brown maple leaf areas, over a pink erythematous background BCC (**d**).

The natural history of BCC often consists of a slowly enlarging macule or papule that does not spontaneously heal and that usually evolves into a nodule or plaque, if not adequately treated. Local tissue infiltration and ulceration are common, unlike systemic involvement. Clinical features of BCC vary widely, and several variants have been described, ranging from superficial to nodular BCC, ulcerating BCC, pigmented BCC, morpheaform BCC, and fibroepithelioma of Pinkus, even though an accurate tumor classification based on clinical and dermoscopic features is not always possible (Figure 2).

Body localizations are consistent with previous UV radiation exposure; according to Gallagher et al., in both sexes, BCCs are more frequently detected on the head, neck, and trunk but leg involvement is twice as frequent in women [50]. Similarly, Pranteda et al. found that among 306 patients affected by BCC from 2008 to 2010, 64.4% were localized on the head, 23.9% in the trunk, 1.9% on the neck, 0.6% on the perineum, 1.3% in upper limbs and 7.9% on legs [51]. BCC usually displays indolent behavior, and it mainly has local infiltrating potential. Due to low reporting, there are few and no accurate literature data on the global incidence of BCC metastasis. Though a higher incidence cannot be excluded, a study conducted in the USA in 2012 reported only 0.04% of metastatic BCC among a cohort of 56,987 patients [52]. Most cases of metastasis originate from sporadic BCC and the primary tumor sites reported to metastasize are the head and neck [53]. The most common metastatic sites are regional lymph nodes followed by hematogenous spread to lung and bone [54]. Unfortunately, metastatic BCC is characterized by a poor prognosis [55] and average survival is reported to be 8–14 months [56,57].

According to the National Comprehensive Cancer Network (NCCN), a series of four tumor characteristics that may predict recurring or metastatic spreading (high-risk tumor) include: (a) localization in high-risk areas like head and neck, (b) lesion diameter (greater or equal to 6 mm) and (c) recurrence after previous treatments. Moreover, (d) histological subtype is a well-established predictor of risk and all cancers with an aggressive growth pattern (micronodular, infiltrative, sclerosing and morpheaform) are more likely to recur than superficial and nodular BCC [58].

Whilst a wide range of local (and mostly curative) treatment modalities is currently available for common BCC “easy-to-treat” (i.e., surgical excision, curettage and electrodesiccation, electrochemotherapy, cryosurgery, topical cytostatic, immunomodulators, photodynamic therapy, and radiotherapy) [59], the management of advanced BCC (aBCC) remains challenging. Both metastatic (mBCC) and locally advanced BCC (laBCC) are included in the advanced BCC category. LaBCCs are defined as large, aggressive, recurrent and deeply invasive tumors not manageable with surgical excision or radiotherapy [60,61]. For example, laBCCs can potentially infiltrate organs such as the eye, nose, and ears and surgical removal can lead to the loss of function of these organs.

Given the complexity of the decision that must be taken in the presence of patients with aBCC, including either laBCC and mBCC, the ultimate treatment choice should be preferably discussed within a multidisciplinary tumor board. Table 1 reports a complete overview of approved and investigative agents for aBCC treatment: specificic indications and mechanisms of action are also discussed.

## 5. Medical Treatment Options for Advanced Disease

### 5.1. Hh Pathway: SMO Inhibitors

The discovery of the role of mutations in the Hh signaling pathway has paved the way for the development of Hh pathway inhibiting agents, such as vismodegib and sonidegib, which have represented a turning point in the aBCC management.

#### 5.1.1. Vismodegib

Vismodegib (Erivedge^®^), an orally administered small molecule, is the first drug to be approved by the FDA and EMA for the treatment of adult patients with mBCC, or with laBCC not eligible for surgery or radiotherapy. Vismodegib is an antagonist of SMO. Through the binding to SMO, whenever PTCH1 is inactivated, the drug is able to block its translocation to the cilium membrane and the downstream activation of the Hh pathway, thus leading to a decrease of proliferation factors and BCC growth.

Approval of vismodegib followed the release of the results of two phase II, open-label, non-comparative, multicenter clinical trials: ERIVANCE BCC and STEVIE [62].

According to the 39-month update results of the ERIVANCE BCC trial, the investigator-assessed objective response rate (ORR) was 48.5% in the mBCC group (all partial responses) and 60.3% in the laBCC group (20 patients had a complete response and 18 patients had a partial response). The median duration of response (DOR) was 14.8 months in the mBCC cohort and 26.2 months in the laBCC group. Although the tolerable safety profile, adverse events (AEs) were common with an incidence ranging from 30 to 70%: the main registered AEs were muscle spasms, alopecia, dysgeusia, weight loss, fatigue, and nausea [62].

Results from the ERIVANCE study were in line with those from the STEVIE trial, which documented a response rate of 68.5% (investigator-assessed) (95% confidence interval [CI)] 65.7–71.3) in patients with laBCC, and 36.9% (95% CI 26.6–48.1) in the metastatic group; the DOR in patients with laBCC was 28.8 months (24.8—Non estimable) and 18.7 months (16.8–21.1) for subgroups with and without Gorlin syndrome; for these latter two subgroups, the DOR in mBCC group was 15.1 (13.9–16.2) and 11.0 (8.3—Nonestimable) [63]. From a study by Fosko et al., it seems that the histopathological subtype (infiltrative, superficial, nodular and keratotic) does not impact BCC response to vismodegib [64].

However, up to 20% of advanced BCC patients develop resistance to vismodegib within the first year of treatment [30]. Brinkhuizen et al. detected two different novel heterozygous missense SMO mutations in two recurrent BCCs [c.842G>T (p.Trp281Leu) in exon 4 and c.961G>A (p.Val321Met) in exon 5, in a 68-year-old subject who had experienced the recurrence of multiple nodules after 20 weeks of treatment with vismodegib. The mutations were absent in the pretreatment tumor tissue, responding tissue, and buccal mucosa of the patient [65]. Furthermore, Sharpe et al. discovered four novel mutations (SMO-T241M, SMO-I408V, SMO-A459V and SMO-C469Y) of SMO associated with drug resistance in patients treated with vismodegib, that experienced regrowth of the tumor; two of these mutations lay in the drug-binding pocket and all conferred resistance to visgmodegib. Moreover, the authors found that vismodegib resistance is associated with concurrent copy number changes in SUFU and GLI2 [38].

#### 5.1.2. Sonidegib

Sonidegib (LDE–225) (Odomzo^®^) is the second oral SMO antagonist, approved by the FDA in 2015 and EMA as a 200 mg oral pill for the treatment of adults with laBCC, not eligible for surgery or radiation therapy, or adults with recurrent laBCC following surgery or radiation therapy.

Sonidegib binds SMO in the drug-binding pocket and acts as an antagonist, preventing the activation of Hh pathway signaling [66].

The efficacy and safety of sonidegib were evaluated in a multicenter, randomized, double-blind, phase 2 BOLT trial, which enrolled patients with a histologically confirmed diagnosis of laBCC not amenable to radiation therapy, curative surgery, or other local therapies, or a histologically confirmed diagnosis of mBCC. Patients were randomized in a 1:2 ratio to receive 200 mg or 800 mg oral sonidegib daily, stratified by disease, histological subtype, and geographical region [67].

LaBCCs were further classified into non-aggressive (nodular and superficial) and aggressive forms (morpheaphorm, infiltrative, micronodular and basosquamous). The 42-month results from the BOLT trial demonstrated that the ORRs were 59.5% and 51.7% (15 of 29) for patients with aggressive and nonaggressive laBCC receiving the 200 mg daily dose and 45.3% and 47.2% for those receiving 800 mg, respectively. Furthermore, in the mBCC group, ORRs were 7.7% (200 mg) and 17.4% (800 mg). The median DOR for responders with laBCC was 26.1 and 23.3 months for the 200 mg and the 800 mg arm respectively. For mBCC patients, the median DOR in the 800 mg arm was not estimable [67]. The most common AEs were similar to those reported with vismodegib and included muscle spasms, alopecia, dysgeusia and nausea. The results from the BOLT trial provided the basis for the approval of sonidegib for the management of laBCC, but not for mBCC.

Danial et al. carried out a clinical trial to assess the response to sonidegib on nine patients with BCC resistant to treatment with vismodegib. None of the patients showed an improvement, on the contrary, some of them experienced a progression of the disease [68]. Indeed, five patients possessed SMO mutations, known from in vitro experiments to confer functional resistance to vismodegib and sonidegib [38,39,69], and three subjects were carriers of SMO mutations not previously known to confer resistance in vitro, thus suggesting that patients with BCCs resistant due to SMO mutations probably are not-responding to SMO inhibitors [68].

#### 5.1.3. Second Generation SMO-Inhibitors

Other drugs belonging to the SMO inhibitor family are currently being evaluated in preclinical and clinical trials.

LEQ506 is a second-generation SMO antagonist, which has been demonstrated to reduce GLI mRNA and to have an IC50 6-fold lower than sonidegib in a human cell line (HEPM). Furthermore, it has exhibited activity against an SMO mutant (D473H) cell line, identified in a medulloblastoma patient who had relapsed after an initial response to vismodegib. In a mouse medulloblastoma allograft model, LEQ506, similarly to the in vitro model, showed an almost complete reduction in GLI mRNA over 24 h after dosing 40 mg/kg. Moreover, mice treated with LEQ506 for 8 days showed a reduction of tumor size similar to sonidegib (85% vs. 90%), but with a plasma and tumor exposure ~3 fold lower due to the higher affinity of LEQ506 to SMO and its lower IC50 compared to sonidegib [70].

LEQ506, given orally, once a day for 21 days, has been evaluated in a phase I clinical trial to assess its safety, tolerability, pharmacodynamic and pharmacokinetic properties in 57 patients affected by laBCC or mBCC, as well as recurrent and refractory medulloblastoma (NCT01106508). From this study, the maximum tolerated dose was 400 mg; mean plasma exposure seemed to increase in a dose-dependent manner from 80 mg to 400 mg. The half-life (T1/2) of LEQ506 was approximately 1 day. The trend of GLI mRNA in treated patients suggested a positive pharmacodynamic response in agreement with the LEQ506 mechanism of action reported in vitro [70]. The drug was generally well tolerated with fatigue as the most common dose-limiting toxicity (DLT) (16.7% in patients treated with 400 mg) and the most common AE. Among all patients, a partial response was observed in 10.5%, stable disease in 26.3% and progressive disease in 42.1% (NCT01106508).

BMS-833923 is a selective SMO antagonist that proved to inhibit GLI1 expression in vitro not only in cell lines expressing wild-type SMO but also in those expressing SMO mutant forms, with and IC50 between 6 and 35 nM. Moreover, the drug inhibited the growth of multiple myeloma (MM) clones and stem cells derived from bone marrow samples from patients with MM [71]. In a phase 1 clinical trial (CA194-002), evaluating the safety of BMS-833923 in patients with metastatic or advanced cancers, two patients with Gorlin syndrome were enrolled. One received 60 mg once every 2 weeks, and the other 300 mg once daily. The first experienced a partial response but a grade 2 drug-related lipase elevation and pancreatitis. All dose cohorts (30 mg, 60 mg, 120 mg, 240 mg) demonstrated a prolonged terminal half-life of BMS-833923 of >7 days. At all doses, a decrease of GLI1 mRNA in skin biopsies was assessed [72].

TAK-441 is another small molecule inhibitor of SMO. In vitro models, the IC50 of GLI transcriptional activity of TAK-441 was 4.4 nM. The in vivo tests, using allograft of medulloblastoma spontaneously occurred in double mutant Ptch1+/− p53−/− transplanted mice, demonstrated a good tumor permeability to TAK-441 and repeated oral administrations of the compound for 14 days resulted in moderate antitumor activity, with a treated/control (T/C) value of 46% at a dose of 1 mg/kg and complete growth inhibition at 25 mg/kg (T/C = 1%) [73].

In the first phase 1 dose-escalation study, 34 patients with different types of solid tumors (21% BCC, 18% colon cancer, 9% colorectal cancer, 9% pancreatic cancer, 6% ovarian cancer, 9% non–small cell lung cancer and 29% other) were enrolled. TAK-441 was administered orally at doses of 50, 100, 200, 400, 800, and 1600 mg per cohort. The maximum feasible dose was 1600 mg; 21% of patients discontinued the treatment due to AEs, but only muscle spasms were considered related to the drug and cerebral hemorrhage was possibly related; 35% of subjects experienced at least 1 serious AE: the most frequent serious AEs were gastrointestinal disorders (12%), neoplasms (progression of underlying disease 12%) and hepatobiliary disorders (9%); 74% of patients experienced drug-related AEs including dysgeusia (47%), muscle spasm (41%) and nausea/vomiting (29%), and the most drug-related AEs were grade 1 or 2. Mean T1/2 ranged from 13.5 to 22.6 h across the dose range. Skin punch biopsies were obtained at baseline and on day 22 to evaluate GLI1 mRNA expression that was strongly reduced (>90%) at all dose levels relative to control genes or SMO. One patient with BCC in the 400 mg cohort experienced a partial response and 7 subjects, including 4 with BCC, belonging to all but the 100 mg cohort, experienced a stable disease [74].

Taladegib (LY2940680) is a SMO antagonist [75] able to bind D473H mutated, vismodegib-resitant SMO [66]. Taladegib has been investigated in a phase I clinical trial where 84 patients were enrolled, including 15 subjects with colon adenocarcinoma and 47 with BCC; of these latter 18 patients had mBCC, 21 laBCC and 8 could not be classified. The trial was divided into three phases: one for the dose-escalation assessment, one for dose confirmation and the last for drug evaluation in BCC patients pre-treated with Hh inhibitor or naїve for any treatment. The maximum tolerable dose (MTD) was 400 mg. For all patients, the most common AEs related to treatment were dysgeusia (48.8%), fatigue (47.6%), nausea (45.2%) and muscle spasms (40.5%). In the BCC expansion cohort, out of 40 patients treated with 400 mg, 30 discontinued due to progressive disease (43.3%), investigator decision (23.3%), AEs (16.6%), and subject decision (16.6%). Overall taladegib demonstrated to be biologically active with a GLI1 mRNA median reduction of 92.3% (interquartile range: 80.9% to 95.7%). Of the 47 BCC patients, 46.8% of patients experienced a complete response or partial response. In the BCC expansion cohort, 31 patients were pre-treated with Hh therapy, and 16 patients were Hh therapy-naїve. Responses defined by the RECIST 1.1 [76] showed promising results both in patients who had not previously received Hh therapy (11/16; 68.8%) and in those who had received prior Hh therapy (11/32; 35.5%) [77].

Itraconazole is an antifungal drug approved by the FDA that turned out to be a potent Hh pathway antagonist. An experimental study conducted by Kim et al. prompted that itraconazole could act downstream of PTCH but not downstream of SMO. Indeed, in cells PTCH−/− and in cells overexpressed for SMO, itraconazole was able to inhibit Hh pathway. However, in transfected cells with constitutively active SmoA1, the inhibitory effect of itraconazole was bypassed. The hypothesis of the authors was that itraconazole could directly act on SMO [78]. Kim et al. provided evidence of the ability of itraconazole to reduce the activity of vismodegib-resistant SMO mutantsThis could be consistent with the supposition of direct binding of itraconazole to SMO, despite in a different site from that of vismodegib. On the other hand, there is not a direct binding assay that can confirm this thesis; therefore, the hypothesis that itraconazole could act on SMO in an indirect manner cannot be completely excluded [79].

In mice with endogenous BCC, itraconazole treatment (100 mg/kg twice per day) was demonstrated to reduce the tumor length, with a rapid tumor re-growth 18 days after the interruption of the drug [78].

Itraconazole was demonstrated to reduce BCC tumor size in humans after 1 month of treatment at 200 mg and to reduce GLI1 mRNA expression in naïve patients but not in patients with prior vismodegib treatment [80]. Ip and McKerrow reported a case of a 69-year-old Caucasian man with mBCC treated with itraconazole 100 mg twice daily that showed a 30% bulk tumor reduction and no evidence of progression [81].

Arsenic trioxide is a drug approved by the FDA for the treatment of acute promyelocytic leukemia, able to inhibit the Hh pathway in mice by binding GLI1 and inhibiting its transcriptional activity [82] and by reducing the ciliary accumulation of GLI2 providing a mechanism of pathway inhibition [83]. Therefore, both itraconazole and arsenic trioxide act to inhibit the Hh pathway in different ways compared to vismodegib and sonidegib.

It was observed that the combination of these two drugs significantly inhibited tumor growth in mice subcutaneous allograft models of BCC compared to control, as well as in vismodegib-resistant murine medulloblastoma allograft models compared to vismodegib and intraconazole or arsenic trioxide alone [79]. Shortly after, an open-label phase 2 trial was conducted in five patients with metastatic resistant-to-SMO BCC treated for three cycles with arsenic trioxide 0.3 mg/kg per day, for 5 days every 28 days and itraconazole 400 mg/day between arsenic trioxide infusion days: despite a GLI1 mRNA reduction in tumor tissue of 75% after drug cycles, no patient experienced a reduction of tumor size and the best overall response was stable disease [84].

### 5.2. Hh Pathway: SMO Independent Inhibitors

#### GLI Inhibitors

Although recent research has mainly focused on therapies selectively targeting the Hh pathway, this approach has shown several limitations such as drug discontinuation due to adverse events as well as primary or secondary resistance due to SMO mutant forms. Moreover, SMO inhibitors are not effective on tumors initiated by downstream mechanisms of SMO, where a non-canonical activation of the Hh pathway occurs. Therefore, compounds able to target directly GLI or modulate it is expected to be promising in the development of cancer therapies.

Currently, a number of GLI inhibitors have been developed and are currently being investigated.

Lauth et al. (2007) selected a hexahydropyrimidine derivative named GANT61 able to inhibit GLI1 and GLI2 transcriptional factors in a dose-dependent manner, inducing a modification of GLI1 that causes an impaired DNA binding [74]. In an NIH 3T3 cell line, stably transfected with a GLI reporter gene, the IC50 of the compound was 5 μM. Also, in mouse embryonic fibroblasts, Ptch1−/−, GANT61 significantly reduced GLI1 mRNA levels at a concentration of 10 μM. Interestingly, other signal transduction pathways, like TNF signaling/NFκB activation, glucocorticoid receptor gene transactivation, and the Ras–Raf–Mek–Mapk cascade were not influenced by treatment with GANT61.

In vivo, using mice injected with GLI1-positive 22Rv1 prostate cancer cells, the treatment with 50 mg/kg of GANT61 induced growth regression until no tumor was palpable and no adverse side effects were observed. After a suspension of drugs for 16 days in four mice, the tumor reappeared in 50% of the animal [85].

Furthermore, in an in vitro setting, Vlčková et al. showed that GANT61 affected the viability of melanoma cell lines inducing apoptosis [86]; these results were confirmed by Réda et al. [87]. So far, no clinical trial in BCC or in other type of tumors have been conducted.

Glabrescione B (GlaB) is a natural compound, found in *Derris glabrescens* (Leguminosae) seeds, directly binding GLI1, thus preventing DNA interaction. The drug was tested in vitro in ASZ001 cell line from mouse BCC to assess the cell death after treatment and the level of GLI1 mRNA; after 72 h of treatment (1 μM, 5 μM, 10 μM) the percentage of cell death was significantly increased compared to the control. Instead, after 48 h at 10 μM, the GLI1 mRNA was approximately 1/5 compared to the control. Notably, in vivo, a significant reduction in tumor growth was observed, as well as GLI1 mRNA levels, in BCC allograft models after 18 days of GlaB administration (100 μmol/kg) compared to the control. GlaB-treated BCC allografts also displayed a decrement of Ki67 and an increment of TUNEL labeling [88].

However, GlaB as it stands is not a good candidate drug due to its poor solubility. Interestingly, Infante et al. found that this problem could be overcome using GlaB micelle-forming amphiphilic polymers that can enhance bioavailability and facilitate biodistribution. This modification could potentially render the agent a promising candidate for the treatment of aBCC, to be further studied in a clinical trial setting [89].

GLI activity can also be modulated by certain tyrosine kinases, such as serine/threonine protein kinases CK2, that are overexpressed in a great number of cancers [90,91].

To date, no mutation of CK2 has been found, but its overexpression helps to maintain an environment susceptible to the development of cancer phenotype [92]. Therefore, CK2 can be considered as a potential target for Hh-driven neoplasms, including BCC.

Silmitasertib (CX-4945), a highly selective CK2 ATP-competitor inhibitor [93], has long been investigated for acute myeloid leukemia (AML) treatment, since in this malignancy CK2 levels are elevated and associated with a poor prognosis [94]. The drug demonstrated in vivo antitumor activity using breast cancer and prostate cancer xenograft models. In breast cancer mice models, CX-4945 exhibited a tumor growth inhibition (TGI) of 88% and 97% when administered twice daily at 25 mg/kg and 75 mg/kg, respectively, and two animals showed a >50% reduction of tumor size in comparison to initial size. In the prostate cancer mice model, the TGI was 93% (75 mg/kg) and in three animals there was no longer evidence of the tumor at the end of the treatment [95]. The drug is currently under investigation in phase I clinical trials on la/mBCC patients and its results can be crucial for establishing the real therapeutic potential of this small molecule (NCT03897036).

### 5.3. BET Inhibitors

Finally, epigenetic alterations of GLI proteins, mediated by certain enzymes called histone deacetylases (HDAC), which remove acetyl groups from GLI, can allow an enhancement of their activity. In this setting, a family of epigenetic enzymes able to prevent the growth of Hh-dependent tumors was recently discovered [96]. The bromodomains and extra-terminal domain (BET) family proteins have been shown to affect GLI transcriptional activity as well as to be involved in cell cycle progression, chromatin compaction, and chemoresistance. BET proteins act by binding acetylated lysines in histones and interacting with the positive transcription elongation factor (p-TEFb), stimulating RNA polymerase II activity and promoting gene expression [97].

BRD4 has been demonstrated to activate Hh signaling in a ligand-independent manner, regulating GLI1- and GLI2-mediated transcription by direct interaction with their promoter [98]. An emerging class of compounds targeting BET was evaluated for Hh-dependent neoplasms [99]. Among them, BRD4 inhibitor mivebresib (ABBV-075) underwent a phase I clinical trial for the treatment of solid tumors (uveal melanoma, colorectal, breast, pancreatic, head and neck, and prostate). Treatment-emergent adverse events occurred in 96% of patients and all patients discontinued the drug principally due to the progression of the disease (74%). No patients experienced a complete or partial response [100].

Another BRD4 inhibitor, NHWD-870, was demonstrated to reduce melanoma cell migration in vitro and the metastasis in vivo mice model [101]. Currently, no data on BCC are available.

### 5.4. Immunotherapy

On 9 February 2021, the FDA approved cemiplimab for the treatment of locally advanced and metastatic BCC. The drug was also approved by EMA in 2023 for the treatment of adult patients with laBCC or mBCC who have progressed on or are intolerant to an Hh inhibitor. Cemiplimab is a monoclonal antibody (mAb) targeting the programmed cell death protein 1 (PD-1) The PD1-PD1 ligand 1 (PDL1) receptor–ligand pair is a dominant immune checkpoint pathway operative in the tumor microenvironment; its normal function in controlling immune homeostasis is induced in cancer cells to evade immune attack [102]. Therefore, mAbs targeting this pathway have emerged as powerful weapons in the oncological armamentarium.

In the first multicenter prospective open-label study with patients with aBCC, the subjects, who had progressed on or were intolerant to previous Hh inhibitors therapy, were treated with cemiplimab 350 mg every 3 weeks for up to 93 weeks or until disease progression or unacceptable toxicity. The authors showed an objective response per independent central review (ICR) in 31% of patients (95% CI 21–42), including 6% complete responses and 25% partial responses. A response rate investigator-assessed of 32% (95% CI 22–43) was reported, including 6% complete responses and 26% partial responses. By ICR, 49% of patients showed stable disease and 11% a progression, with Kaplan–Meier estimated duration of response exceeding 1 year in 85% (61–95) of responders. By ICR, the median time of response was 4.3 months (IQR 4.2–7.2). Grade 3–4 treatment-emergent AEs occurred in 48% of patients with the most common being hypertension and colitis and fatigue, urinary tract infection, and visual impairment; 11% of patients discontinued treatment due to treatment-related AEs. The most common serious treatment-related AEs were colitis in 4% of patients and adrenal insufficiency in 2% [103].

From the same study, an interim analysis of 28 patients with mBCC showed an objective response investigator-assessed in 28.6% (95% CI 13.2–48.7) of patients, with an observed DOR of 9–23 months. By ICR, the median time to respond was 3.2 months (range 2.1–10.5). Like patients with aBCC, the most severe AE (grade > 3) was hypertension [104]. The median Kaplan-Meyer estimation of progression-free survival was 8.3 months and of overall survival was 25.7 months.

Cemiplimab, administered at a dosage of 350 mg every 3 weeks, has also been used to treat BCC with pulmonary metastasis in a 66-year-old male Caucasian patient who had developed severe toxicity to vismodegib. After approximately 12 months of therapy, a partial remission of the lesion present in the left lung with lower fluorodeoxyglucose (FDG) uptake was observed and the general health state of the patient was improved. However, after three months of discontinuation of therapy, an increment in tumor size was noticed [105].

## 6. Discussion

Along with the progressive population aging and the photodamage effects of the last 50 years, the BCC burden has exponentially grown and this trend will continue with relevant sanitary costs and increasing waiting lists.

Localized BCC is usually considered an “easy to treat” lesion. It can be cured with different therapeutic strategies such as surgical excision, curettage, cryosurgery, topical cytostatics, photodynamic therapy and radiotherapy.

Conversely, laBCC and mBCC require a more complex intervention and a multidisciplinary approach including systemic treatments with immunomodulating or chemiotherapeutic action.

Systemic chemotherapy, mainly consisting of platinum-based regimens, has been the only therapeutic option in the past decades but its efficacy has been addressed only in case reports and case series. The lack of randomized trials and the toxicity profile suggest that chemotherapy should be considered as an option for second-line treatment in selected, fit patients.

The discovery of the Hh pathway and the release of vismodegib and sonidegib, have changed dramatically the management of BCC non-amenable to local treatment. Since the Hh signaling pathway is a key driver in the biology of BCC, its selective antagonism provides a range of promising treatment options for patients with laBCC or mBCC. Vismodegib and sonidegib were the first FDA and EMA-approved drugs for aBCC. Unfortunately, a significant proportion of patients either do not respond to these drugs or develop drug resistance, often requiring a switch to an alternative agent or a combination therapy. Besides, severe side effects are common, and many patients require early treatment discontinuation. For these reasons, these agents are unlikely to be the answer for long-term management of aBCC, and the choice of further lines of treatment is still an open issue.

Checkpoint inhibitors such as cemiplimab were recently approved by the FDA and EMA and may represent an encouraging option for aBCC in both treatment naïve patients as well as in patients who have progressed on Hh inhibitors. According to our clinical experience so far, the safety profile of cemiplimab appears to be more reassuring than that of Hh inhibitors.

Nowadays, there are several novel molecules under investigation for the treatment of aBCC that hold the promise to expand the current therapeutic armamentarium and overcome the drug resistance often observed in clinical practice. These molecules are second-generation SMO inhibitors that target components downstream of SMO and can be efficacious for the treatment of cancers linked to the GLI aberrant activation, including BCC.

Among SMO inhibitors, LEQ506, TAK-441 and taladegib demonstrated promising results in phase 1 clinical trials. In detail, taladegib was shown to be effective in patients who had received prior Hh therapy.

Regarding the GLI inhibitors, only silmitaserib is going through a phase I clinical trial for the treatment of aBCC. The results are not yet available, but the evidence reported in vivo studies for breast and prostate cancers are encouraging. GANT61 and glabrescione B are being investigated in a pre-clinical phase, showing GLI downregulation ability in in vitro and in vivo models. Lastly, BET inhibitors have recently shown promising results against Hh-dependent neoplasms. Although the data on these novel agents are overall encouraging, further investigation is necessary. For example, there is still no evidence on mivebresib for aBCC treatment. In a clinical trial on solid tumors, no patients experienced a complete or partial response and a high percentage of them discontinued the drug due to the progression of the disease.

In this flourishing field of development of new therapies, translational research is called to give its fundamental contribution to the evaluation of novel molecules for the treatment of aBCC. However, the experiments on primary cell cultures isolated from human BCC have often proved unsuccessful due to the difficulties in stabilizing this type of cancer cells in an in vitro setting [106,107]. In this context, the use of ex-vivo cultures of skin explants from human BCC [108] could address this gap and help researchers understand biological processes underlying BCC development and growth as well as represent a valid model for testing therapeutic agents.

In conclusion, the discovery of new drugs and the development of new tumor culture techniques for their evaluation will likely play a crucial role in addressing the current unmet clinical need in aBCC.

## Figures and Tables

**Table 1:** Approved and under investigation agents for the treatment of aBCC.

Compound	Type of Molecule	Indication	Stage of Approval for BCC Treatment	Mechanism of Action
Vismodegib	Small Molecule	aBCC	Approved	SMO inhibitor
Sonidegib	Small Molecule	aBCC	Approved	SMO inhibitor
LEQ506	Small molecule	aBCC	Phase I	SMO inhibitor
BMS-833923	Small molecule	Advanced or metastatic solid tumors	Phase I	SMO inhibitor
TAK-441	Small molecule	Advanced nonhematologic malignancies	Phase I	SMO inhibitor
Taladegib	Small molecule	Colon adenocarcinoma and BCC	Phase I	SMO inhibitor
Itraconazole	Azole	aBCC	Phase II	Hh inhibitor
GANT61	Small molecule	GLI activation dependent tumors	Pre-clinical phase	GLI inhibitor
Glabrescione B	Natural compound found in *Derris glabrescens*	GLI activation dependent tumors	Pre-clinical phase	GLI inhibitor
Silmitasertib	Small molecule	aBCC	Phase I	CK2 inhibitor
Mivebresib	Small molecule	Solid tumors	Pre-clinical phase	BET inhibitor
NHWD-870	Small molecule	GLI activation dependent tumors	Pre-clinical phase	BET inhibitor
Cemiplimab	Monoclonal antibody	aBCC	Approved	PD-1 inhibitor
